# Discrepancy Between Clinical and Pathologic Nodal Status of Esophageal Cancer and Impact on Prognosis and Therapeutic Strategy

**DOI:** 10.1245/s10434-017-6088-8

**Published:** 2017-09-25

**Authors:** Sheraz R. Markar, Caroline Gronnier, Arnaud Pasquer, Alain Duhamel, Hélène Behal, Jérémie Théreaux, Johan Gagnière, Gil Lebreton, Cécile Brigand, Florence Renaud, Guillaume Piessen, Bernard Meunier, Denis Collet, Christophe Mariette, on Behalf of the FREGAT Working Group—FRENCH—AFC, Guillaume Luc, Magalie Cabau, Jacques Jougon, Bogdan Badic, Patrick Lozach, Issam El Nakadi, Serge Cappeliez, Gil Lebreton, Jean Lubrano, Arnaud Alves, Renaud Flamein, Denis Pezet, Federica Pipitone, Bogdan Stan Iuga, Nicolas Contival, Eric Pappalardo, Styliani Mantziari, Nicolas Demartines, Flora Hec, Marguerite Vanderbeken, Sébastien Degisors, Hélène Marin, Fabien Fredon, Alain Gainant, Muriel Mathonnet, Jean-Marc Bigourdan, Salim Mezoughi, Jean-Yves Mabrut, Christian Ducerf, Oussama Baraket, Gilles Poncet, Delphine Vaudoyer, Peggy Jourdan Enfer, Laurent Villeneuve, Olivier Glehen, Thibault Coste, Jean Michel Fabre, Frédéric Marchal, Romain Frisoni, Ahmet Ayav, Laurent Brunaud, Laurent Bresler, Charlotte Cohen, Olivier Aze, Nicolas Venissac, Daniel Pop, Jérôme Mouroux, Ion Donici, Michel Prudhomme, Emanuele Felli, Stéphanie Lisunfui, Marie Seman, Gaelle Godiris Petit, Mehdi Karoui, Christophe Tresallet, Fabrice Ménégaux, Brice Malgras, Denis Lantuas, Karine Pautrat, Marc Pocard

**Affiliations:** 1 Department of Surgery and Cancer, Imperial College, London, UK; 2 Department of Digestive and Oncological Surgery, Univ.Lille, Claude Huriez University Hospital, Lille, France; 3 Univ.Lille, UMR-S 1172 – JPARC – Centre de Recherche Jean-Pierre AUBERT Neurosciences et Cancer, Lille, France; 4 Inserm, UMR-S 1172, Lille, France; 5 Department of Digestive Surgery of Edouard, Herriot University Hospital, Lyon, France; 6 SIRIC OncoLille, Lille, France; 7 Department of Biostatistics, Univ.Lille, University Hospital, Lille, France; 8 Cavale Blanche University Hospital, Brest, France; 9 Estaing University Hospital, Clermont-Ferrand, France; 10 Côte de Nacre University Hospital, Caen, France; 11 Hautepierre University Hospital, Strasbourg, France; 12 Department of Pathology, Univ.Lille, University Hospital, Lille, France; 13 France Pontchaillou University Hospital, Rennes, France; 14 Haut-Levêque University Hospital, Bordeaux, France; 15 Department of Digestive and Oncological Surgery, University Hospital Claude Huriez–Regional University Hospital Center, Lille Cedex, France

## Abstract

**Background:**

The impact of discrepancies between clinical (c) and pathologic (p) stages of esophageal cancer remains a poorly understood issue. This study aimed to compare the prognosis of patient groups treated by primary surgery including clinical N0/pathologic N0 (cN0pN0), clinical N0/pathologic N+ (cN0pN+), clinical N+/pathologic N0 (cN+pN0), and clinical N+/pathologic N+ (cN+pN+).

**Methods:**

Data were collected from 30 European centers during the years 2000 to 2010. Among 2944 recruited patients, 1554 patients receiving primary surgery met the inclusion criteria including 613 cN0pN0, 403 cN0pN+, 220 cN+pN0, and 318 cN+pN+ patients. Analyses with adjustment of the propensity score were used to compensate for differences in baseline characteristics.

**Results:**

Clinical T stages 3 and 4 were increased in cN+pN+ (73.0%), cN0pN+ (49.6%), and cN+pN0 (51.8%) compared with cN0pN0 (32.8%). Compared with cN0pN0, cN+pN+ and cN0pN+ showed an increase in the proportion of adenocarcinoma histologic subtype, poor tumor differentiation, pathologic T3 and T4 stages, and R1/2 resection margin. Adjusted 5-year overall survival (hazard ratio [HR] 3.12; 95% confidence interval [CI] 2.57–3.78; *P* < 0.001) and event-free survival (HR 2.87; 95% CI 2.39–3.45; *P* < 0.001) were significantly reduced in cN0pN+ compared with cN0pN0. No significant differences in 5-year overall survival or event-free survival between cN0pN+ and cN+pN+ were observed. Regression analysis identified an association of distal tumor location, advanced clinical T stage, and poor tumor differentiation with pN+ disease.

**Conclusions:**

This large multicenter study showed that cN0pN+ has a prognosis similar to that of cN+pN+ and worse than that of cN0pN0. Patients with clinical N0 disease but risk factors for pathologic N+ disease may benefit from neoadjuvant therapy before surgery.

In recent years, the treatment methods available for esophageal cancer have increased substantially.^1^^–^ 3 Paralleling this growth in treatment options has been a growth in evidence for patient- and tumor-specific strategies.^4^^–^ 6 However current guidelines are limited by the quality of the available evidence for individual clinical stages and histologic subtypes of esophageal cancer, which often are grouped together in publications to increase the statistical power of the study at the cost of creating heterogeneous analyses. Consequently, ensuring interpretation of results remains challenging.^7^^–^ 9

Treatment of clinical N0/pathologic N+ (cN0pN+) esophageal cancer remains a relatively controversial and poorly understand issue. Adjuvant therapy for patients with pN+ esophageal cancer has limited efficacy in improving long-term prognosis.^10^ Therefore, many clinicians have advocated for the use of neoadjuvant therapy in the treatment of patients with cT2/3 N0 esophageal cancer due to the 20% of pN+ disease in esophageal cancers with submucosal invasion.^11^,^12^ However, it may be perceived that this is a highly aggressive and unnecessary treatment approach because the prognosis for cN0pN+ has previously not been compared with clinical N+/pathologic N+ (cN+pN+) or clinical N0/pathologic N0 (cN0pN0). Therefore, it may be suggested that cN0pN+ has an intermediate prognosis between these other two groups and therefore deserves individual consideration.

The primary objective of the current study was to compare the long-term prognosis for cN0pN+ patient groups treated by primary surgery with cN0pN0, clinical N+/pathological N0 (cN+pN0), and cN+pN+ patient groups.

The secondary objectives were to compare the long-term prognosis of cN0pN+ patients in subset comparisons for histologic subtype and T stage, to evaluate the prognostic effect of adjuvant therapy in cN0pN+ patients, and to identify risk factors for pN+ status in cN0 patients.

## Methods

### Patient Eligibility Criteria

A dedicated website (http://www.chirurgie-viscerale.org), was used to capture data from 2944 consecutive adult patients undergoing surgical resection for esophageal cancer (including Siewert types 1 and 2 junctional tumors) with curative intent in 30 French-speaking European centers between 2000 and 2010. An independent team monitored and audited the data capture to minimize missing data and to ensure both concordance and inclusion of consecutive patients.

Patient malnutrition was defined by weight loss of more than 10% during a 6-month period before surgery. High-volume centers were defined as those performing more than eight resections per year during the 10-year study period.^13^

As recommended by French national guidelines,^14^ the approach to clinical staging used a combination of endoscopic ultrasound (EUS) for transversable tumors, computerized tomography (CT), and, on demand, positron emission tomography (PET). The study was accepted by the regional institutional review board on 15 July 2013, and the database was registered in the Clinicaltrials.gov website under the identifier NCT 01927016.

### Data Collection

Patient demographics and tumor-related data were collected. Complications were defined on the basis of the definitions used in the MIRO trial protocol.^15^ Histologic staging of tumors was based on the 7th edition of the Union Internationale Contre le Cancer (UICC)/TNM classification.^16^

### Inclusion Criteria

From the 2944 consecutive surgically treated patients collected in the database, we excluded those treated with neoadjuvant therapy (*n* = 1358) and those with metastatic disease (*n* = 18) or synchronous cancer at diagnosis (*n* = 14), leaving 1554 patients. The treatment approach for individual patients was decided at the local center with multidisciplinary team meetings for all the participating centers.

### Follow-Up Evaluation: Survival and Recurrence

During follow-up period, clinical examination and thoracoabdominal CT every 6 months for 5 years was recommended, with upper gastrointestinal endoscopy at 2 years.^14^ In cases of suspected recurrence, thoracoabdominal CT scan and upper gastrointestinal endoscopy were performed. Histologic, cytologic, or unequivocal radiologic proof was required before a diagnosis of recurrence was determined, and using this, disease-free survival was calculated.

### Statistical Analysis

Statistical analysis was performed using SPSS version 20.0 software (SPSS, Chicago, IL, USA) or the SAS software package, release 9.3 (SAS Institute, Cary, NC, USA). Data are presented as number (%) or median (range). Comparison of patient demographics, surgical technique, tumor pathology, and postoperative outcomes between the four study groups was performed using the Kruskall–Wallis test for quantitative variables or the Chi square test (Fisher’s exact test was used when expected cell frequencies were lower than 5) for categorical variables.

Overall and disease-free survivals were estimated using the Kaplan–Meier method and compared between the four study groups using the log-rank test. We further compared the overall and disease-free survivals between cN0pN+ and each of the other subgroups using Cox’s proportional hazard model, and hazard ratios for cN0/pN+ relative to each of the other subgroups were calculated as effect sizes. Proportional hazard assumption was checked using the Schoenfeld residuals.

To reduce the effects of potential confounding factors [study period, age, gender, American Society of Anesthesiologists (ASA) score, malnutrition, center volume, clinical T stage, tumor location, surgical technique, histologic subtype, adjuvant therapy] in the analysis of the short- and long-term outcomes between cN0/pN+ and cN0/pN0, and between cN0/pN+ and cN+/pN+, we calculated a propensity score for each comparison. The propensity scores were estimated using a multivariable logistic regression model, with study groups as the dependent variable and potential confounding factors as the independent variables.

To avoid case deletion in propensity score adjustment analyses due to missing information for malnutrition (22%), missing values for malnutrition were imputed by multiple imputations using all variables included in propensity score calculations (including the study groups).^17^ Missing data were imputed under a missing-at-random assumption by using a regression-switching approach (chained equation with *m* = 10 imputations obtained using the R Statistical Software, version 3.03 (R Development Core Team, Auckland, New Zealand), with a predictive mean matching method for continuous variables, a logistic regression model for binary variables, and an ordinal logistic regression model for ordinal categorical variables.^18^

In each imputed data set, propensity score-adjusted analyses were performed using logistic regression models for short-term outcomes and Cox’s proportional hazard models for long-term outcomes. Logistic and Cox’s regressions estimates obtained in the different imputed data sets were combined using Rubin’s rules.^19^ Adjusted odds ratios (ORs) and hazard ratios (HRs) with 95% confidence intervals (CIs) were derived from these combined estimates as effect size measurements (using cN0/pN0 and cN+/pN+ as reference groups).

Exploratory analyses evaluating survival according to the histologic subtype and clinical T stage and the impact of adjuvant treatment in pN+ groups were performed. Finally, among the cN0 patients, we studied the factors associated with pN+ disease in bivariate and multivariate analyses. Variables associated with pN+ disease in bivariate analyses (*P* < 0.05) were introduced into a multivariable logistic regression model.

All statistical tests were two-sided, with the threshold of significance set at a *P* value lower than 0.05.

## Results

### Overall Population Characteristics

From the original data set of 2944 patients, 1610 patients did not meet the inclusion criteria (reasons described in the Methods section), leaving 1554 patients who received primary surgery and were included in this study. Clinical tumoral staging was based on CT scan for 100% of the patients, combined with endoscopic ultrasound for 73% of the patients (not transversable tumor for 15%, not performed for 12%) and PET scan for 47% of the patients. The majority of the patients were 60 years of age or older (55%), had an ASA grade of 2 (57.7%), and had undergone surgery in a high-volume center (61.0%).

Squamous cell carcinoma was diagnosed for 719 patients (46.3%) and adenocarcinoma for 835 patients (53.7%). The median number of lymph nodes harvested was 16 (range 3–72), and the incidence of a R1/2 resection margin was 11.4% (*n* = 177). The four patient groups treated by primary surgery and included in the study were cN0pN0 (*n* = 613), cN0pN+ (*n* = 403), cN+pN0 (*n* = 220), and cN+pN+ (*n* = 318).

### Comparison of Patient Demographics, Clinical and Pathologic Staging, and Outcomes Between Groups

The groups did not differ significantly in terms of baseline patient demographics, except for the proportion of malnutrition, which was increased in the cN+pN+ group (21.4%), the cN0pN+ group (19.1%), and the cN+pN0 group (13.6%) compared with the cN0pN0 group (7.3%) (Table 1). The incidence of clinical T stage 3 or 4 was increased in the cN+pN+ group (73%), the cN0pN+ group (49.6%), and the cN+pN0 group (51.8%) compared with the cN0pN0 group (32.8%). The cN+pN+ and cN0pN+ groups had a significantly greater proportion of lower third esophageal tumors than the cN0pN0 and cN+pN0 groups. This was reflected in differing surgical techniques and increased use of the Ivor Lewis technique in the cN+pN+ and cN0pN+ groups.

**Table 1:** Comparison of patient demographics and surgical technique according to clinical (cN)/pathologic (pN) node groups

Variable	Overall incidence (*n* = 1554) *n* (%)	cN0pN0 (*n* = 613) *n* (%)	cN0pN+ (*n* = 403) *n* (%)	cN+pN0 (*n* = 220) *n* (%)	cN+pN+ (*n* = 318) *n* (%)	*P* value
Surgery after 2006^a^	614 (39.5)	269 (43.9)	143 (35.5)	101 (45.9)	101 (31.8)	<0.001
Age ≥ 60 years^a^	855 (55.0)	330 (53.8)	218 (54.1)	137 (62.3)	170 (53.5)	0.140
Male incidence^a^	1256 (80.8)	484 (79.0)	330 (81.9)	171 (77.7)	271 (85.2)	0.072
ASA score^a^						0.915
1	240 (15.4)	99 (16.2)	60 (14.9)	32 (14.5)	49 (15.4)	
2	897 (57.7)	360 (58.7)	235 (58.3)	126 (57.3)	176 (55.3)	
3	398 (25.6)	147 (24.0)	101 (25.1)	60 (27.3)	90 (28.3)	
4	19 (1.2)	7 (1.1)	7 (1.7)	2 (0.9)	3 (0.9)	
Malnutrition^a^	220 (14.2)	45 (7.3)	77 (19.1)	30 (13.6)	68 (21.4)	<0.001
Center volume (≥8/year)^a^	948 (61.0)	371 (60.5)	251 (62.3)	129 (58.6)	197 (61.9)	0.705
Clinical T category^a^						<0.001
1	389 (25.0)	265 (43.2)	68 (16.9)	44 (20.0)	12 (3.8)	
2	418 (26.9)	147 (24.0)	135 (33.5)	62 (28.2)	74 (23.3)	
3	382 (24.6)	68 (11.1)	121 (30.0)	55 (25.0)	138 (43.4)	
4	365 (23.5)	133 (21.7)	79 (19.6)	59 (26.8)	94 (29.6)	
Esophageal tumor location^a^						<0.001
Upper	224 (14.4)	112 (18.3)	34 (8.4)	48 (21.8)	30 (9.4)	
Middle	509 (32.8)	227 (37.0)	124 (30.8)	74 (33.6)	84 (26.4)	
Lower	821 (52.8)	274 (44.7)	245 (60.8)	98 (44.5)	204 (64.2)	
Surgical technique^a^						0.010
Ivor Lewis	1098 (70.7)	413 (67.4)	310 (76.9)	148 (67.3)	227 (71.4)	
3 stage	161 (10.4)	67 (10.9)	29 (7.2)	33 (15)	32 (10.1)	
Transhiatal	295 (19.0)	133 (21.7)	64 (15.9)	39 (17.7)	59 (18.6)	

*ASA* American society of anesthesiologists

^a^Included in the propensity-matched analysis

Tumor pathology differed substantially between the groups (Table 2). Compared with the cN0pN0 and cN+pN0 groups, the cN+pN+ and cN0pN+ groups showed an increase in the proportion of adenocarcinoma histologic subtype, poor tumor differentiation, pathologic T3 or T4 stage, pathologic stage 3 disease, and R1/2 resection margin. The number of lymph nodes harvested was marginally greater for cN+pN+ (*n* = 20; range 3–72), cN0pN+ (*n* = 17; range 3–49), and cN+pN0 (*n* = 16; range 3–48) than for cN0pN0 (*n* = 13; range 3–70). The median number of positive lymph nodes was 3 for the cN+pN+ group and 2 for the cN0pN+ group. The groups did not differ significantly in short-term mortality or morbidity (Table 2).

**Table 2:** Comparison of tumor pathology and postoperative outcomes according to clinical (cN)/pathologic (pN) node groups

Variable	Overall incidence (*n* = 1554) *n* (%)	cN0pN0 (*n* = 613) *n* (%)	cN0pN+ (*n* = 403) *n* (%)	cN+pN0 (*n* = 220) *n* (%)	cN+pN+ (*n* = 318) *n* (%)	*P* value
Histologic subtype						<0.001
Squamous cell cancer	719 (46.3)	302 (49.3)	176 (43.7)	122 (55.5)	119 (37.4)	
Adenocarcinoma	835 (53.7)	311 (50.7)	227 (56.3)	98 (44.5)	199 (62.6)	
Tumor differentiation						<0.001
Good	533 (34.3)	237 (38.7)	115 (28.5)	86 (39.1)	95 (29.9)	
Average	540 (34.7)	185 (30.2)	175 (43.4)	64 (29.1)	116 (36.5)	
Poor	239 (15.4)	60 (9.8)	72 (17.9)	29 (13.2)	78 (24.5)	
Data missing	242 (15.6)	131 (21.4)	41 (10.2)	41 (18.6)	29 (9.2)	
pT category						<0.001
pT0/1	631 (40.6)	416 (67.9)	73 (18.1)	108 (49.1)	34 (10.7)	
pT2	267 (17.2)	82 (13.4)	89 (22.1)	36 (16.4)	60 (18.9)	
pT3	575 (37.0)	102 (16.6)	211 (52.4)	68 (30.9)	194 (61.0)	
pT4	81 (5.2)	13 (2.1)	30 (7.4)	8 (3.6)	30 (9.4)	
pN category						<0.001
pN0	833 (53.6)	613 (100)	0 (0)	220 (100)	0 (0)	
pN1	352 (22.7)	0 (0)	212 (52.6)	0 (0)	140 (44.0)	
pN2	208 (13.4)	0 (0)	117 (29.0)	0 (0)	91 (28.6)	
pN3	161 (10.4)	0 (0)	74 (18.4)	0 (0)	87 (27.4)	
pTNM stage						<0.001
0	59 (3.8)	47 (7.7)	0 (0)	12 (5.5)	0 (0)	
I	583 (37.5)	451 (73.6)	0 (0)	132 (60.0)	0 (0)	
II	328 (21.1)	102 (16.6)	108 (26.8)	68 (30.9)	50 (15.7)	
III	584 (37.6)	13 (2.1)	295 (73.2)	8 (3.6)	268 (84.3)	
Resection margin						<0.001
R0	1377 (88.6)	576 (94.0)	347 (86.1)	197 (89.5)	257 (80.8)	
R1/R2	177 (11.4)	37 (6.0)	56 (13.9)	23 (10.5)	61 (19.2)	
Lymph nodes harvested	16 (3–72)	13 (3–70)	17 (3–49)	16 (3–48)	20 (3–72)	<0.001
Positive lymph nodes	0 (0–32)	0 (0–0)	2 (1–32)	0 (0–0)	3 (1–32)	<0.001
In-hospital mortality	109 (7.0)	45 (7.3)	21 (5.2)	19 (8.6)	24 (7.5)	0.371
In-hospital morbidity	905 (58.2)	364 (59.4)	220 (54.6)	134 (60.9)	187 (58.8)	0.359
Reintervention	245 (15.8)	101 (16.5)	59 (14.6)	41 (18.6)	44 (13.8)	0.411
Adjuvant therapy (any)	267 (17.2)	28 (4.6)	135 (33.5)	13 (5.9)	91 (28.6)	<0.001
Chemoradiotherapy	136 (8.8)	14 (2.3)	63 (15.6)	7 (3.2)	52 (16.4)	<0.001
Chemotherapy	105 (6.8)	6 (1.0)	66 (16.4)	4 (1.8)	29 (9.1)	
Radiotherapy	26 (1.7)	8 (1.3)	6 (1.5)	2 (0.9)	10 (3.1)	

*pTNM* pathologic tumor-node-metastasis

### Unadjusted Long-Term Analysis

The unadjusted 5-year overall survival (23.3% vs 67.0%; HR 3.15; 95% CI 2.62–3.79; *P* < 0.001) and the event-free survival (20.7% vs 61.6%; HR 2.91; 95% CI 2.44–3.47; *P* < 0.001) were significantly reduced in the cN0pN+ group compared with the cN0pN0 group. Also, the findings showed significant increases in overall recurrence (64.6% vs 19.0%; HR 5.16; 95% CI 3.94–6.75; *P* < 0.001), locoregional recurrence (24.9% vs 9.9%; HR 3.00; 95% CI 1.98–4.55; *P* < 0.001), distant recurrence (28.6% vs 6.1%; HR 5.64; 95% CI 3.52–9.05; *P* < 0.001), and mixed recurrence (20.1% vs 3.5%; HR 5.95; 95% CI 3.37–10.50; *P* < 0.001) in the cN0pN+ group compared with the cN0pN0 group.

**Fig. 1:**
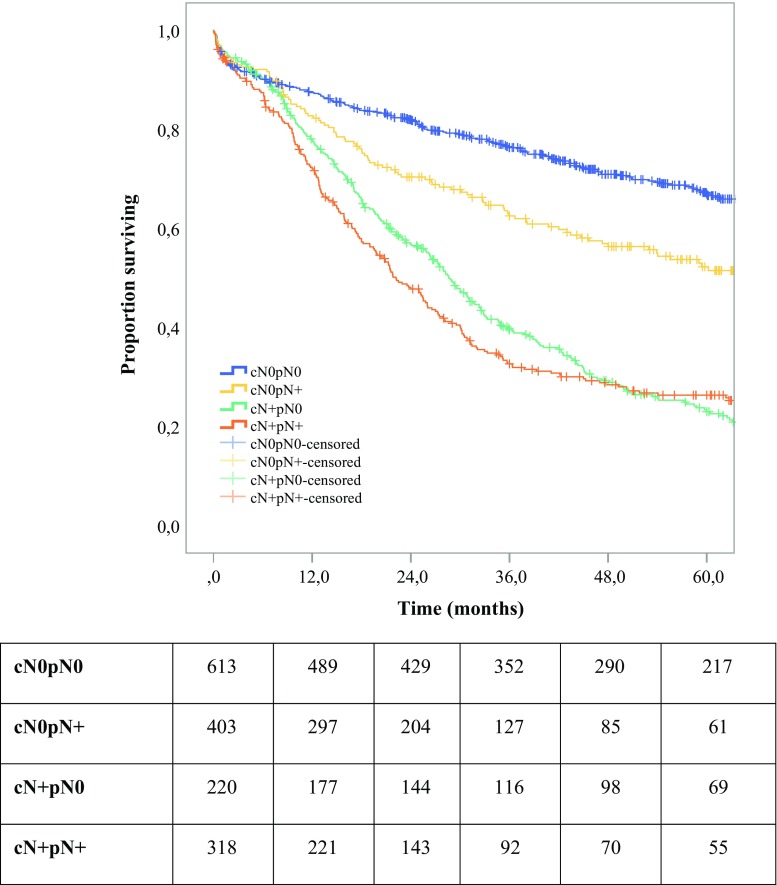
Unadjusted comparison of 5-year overall survival between four groups: cN0pN0, cN0pN+, cN+pN0, and cN+pN+ (*P* < 0.001)

However, the findings showed no significant differences in overall survival (23.3% vs 26.4%; HR 0.92; 95% CI 0.77–1.09; *P* = 0.331) or event-free survival (20.7% vs 20.4%; HR 0.84; 95% CI 0.71–1.00; *P* = 0.050) between the cN0pN+ and cN+pN+ groups (Fig. 1). The findings also showed no significant differences between these groups in overall recurrence (64.6% vs 68.3%; HR 0.83; 95% CI 0.66–1.04; *P* = 0.104), locoregional recurrence (24.9% vs 29.0%; HR 0.79; 95% CI 0.53–1.17; *P* = 0.235), distant recurrence (28.6% vs 30.6%; HR 0.84; 95% CI 0.59–1.21; *P* = 0.355), or mixed recurrence (20.1% vs 18.9%; HR 0.97; 95% CI 0.63–1.50; *P* = 0.889).

### Adjusted Comparison of cN0pN+ and cN0pN0 Patients

After adjustment on the propensity score, cN0pN+ and cN0pN0 did not differ significantly in terms of in-hospital mortality or morbidity. Importantly, the cN0pN+ group showed an increased incidence of R1/R2 resection margin (OR 1.62; 95% CI 1.29–2.05; *P* < 0.001). The 5-year overall survival (HR 3.12; 95% CI 2.57–3.78; *P* < 0.001) and event-free survival (HR 2.87; 95% CI 2.39–3.45; *P* < 0.001) were significantly reduced in the cN0pN+ group. The cN0pN+ group showed significant increases in overall recurrence (HR 4.74; 95% CI 3.58–6.27; *P* < 0.001), locoregional recurrence (HR 3.04; 95% CI 1.97–4.69; *P* < 0.001), distant recurrence (HR 4.70; 95% CI 2.86–7.71; *P* < 0.001), and mixed recurrence (HR 5.60; 95% CI 3.10–10.12; *P* < 0.001).

### Adjusted Comparison of cN0pN+ and cN+pN+ Patients

After adjustment on the propensity score, cN0pN+ and cN+pN+ did not differ significantly in terms of in-hospital mortality or morbidity, or in terms of R1/R2 resection margins. The groups did not differ significantly in terms of 5-year overall survival (HR 0.94; 95% CI 0.78–1.12; *P* = 0.467) or event-free survival (HR 0.87; 95% CI 0.73–1.04; *P* = 0.121). The groups also did not differ significantly in terms of overall recurrence (HR 0.88; 95% CI 0.69–1.11; *P* = 0.268), locoregional recurrence (HR 0.83; 95% CI 0.55–1.24; *P* = 0.368), distant recurrence (HR 0.89; 95% CI 0.61–1.30; *P* = 0.549), or mixed recurrence (HR 0.97; 95% CI 0.62–1.52; *P* = 0.893).

### Adjusted Survival Analyses According to Histologic Subtype and Clinical T Stage (Appendix 1)

Subset analysis according to the histologic subtype showed that cN0pN+ had a significantly reduced 5-year overall survival (*P* < 0.001) and event-free survival (*P* < 0.001) compared with cN0pN0 in both the squamous cell and adenocarcinoma subgroups. However, cN0pN+ showed no significant difference in 5-year overall survival (*P* > 0.108) or event-free survival (*P* > 0.226) compared with cN+pN+ in each histologic subtype.

Subset analysis according to clinical T stage showed that cN0pN+ had a significantly reduced 5-year overall survival (*P* < 0.011) and event-free survival (*P* < 0.010) compared with cN0pN0 for all clinical T1, T2, T3, and T4 stages. Compared with cN+pN+ in each clinical T stage, cN0pN+ showed no significant difference in 5-year overall survival (*P* > 0.147) or event-free survival (*P* > 0.139).

### Impact of Adjuvant Treatment in pN+ Groups

The use of adjuvant therapy was increased in pathologically node-positive groups (Table 1), but without offering any 5-year survival benefit (24.0% vs 26.6%; HR 0.85; 95% CI 0.70–1.03; *P* = 0.101).

### Risk Factors for pN+ Disease for cN0 Patients

Univariable comparison of the pN+ and pN0 groups demonstrated significant increases in the pN+ group in the proportion of males (81.9% vs 79.0%; *P* = 0.018), tumors in the lower third (62.3% vs 44.7%; *P* < 0.001), malnourished patients (20.1% vs 9.0%; *P* < 0.001), clinical T3/4 tumors (59.9% vs 37.8%; *P* < 0.001), adenocarcinoma histologic subtype (59.1% vs 49.1%; *P* < 0.001), and poor tumor differentiation (20.8% vs 10.7%; *P* < 0.001). Regression analysis-confirmed variables independently associated with pN+ disease included lower third tumor location (OR 3.11; 95% CI 2.13–4.54; *P* < 0.001), advanced clinical T (T3/T4) stage (OR 7.70; 95% CI 5.48–10.82; *P* < 0.001), and poor tumor differentiation (OR 2.13; 95% CI 1.51–3.00; *P* < 0.001).

## Discussion

The results of this large retrospective multicenter European study suggest that the long-term prognosis for cN0pN+ esophageal cancer patients is significantly worse than for cN0pN0 patients, but similar to that for cN+pN+ patients. Adjuvant therapy failed to alter the prognosis in the cN0pN+ group. Variables associated with pN+ disease included lower third tumor location, advanced clinical T stage, and poor tumor differentiation, suggesting a high-risk cohort of cN0 patients who may benefit from neoadjuvant therapy before surgery.

Important limitations must be considered when the results from this study are interpreted, including its retrospective observational design. To minimize any bias associated with data collection methods during this study, an independent monitoring team audited the data capture to minimize missing data, to control concordance, and to ensure inclusion of consecutive patients. Despite these steps, 22% of the cases in the propensity-matched analysis required imputing of data, which may have led to the introduction of bias. However, this effect was limited by the use of random assumption and a regression-switching approach to imputation.

Furthermore, despite analysis and control for many important factors that can influence long-term survival and cancer recurrence using propensity score-matched and multivariable analyses, other potential confounding variables exist that were not studied. Preoperative tumoral staging quality could be questioned, but the approach to clinical staging was similar between all the study centers, and as described by the French national guidelines, used a combination of endoscopic ultrasound for tranversable tumors, CT scans, and, on demand, PET. In addition, such a large multicentric study reflects most common clinical practice and probably a high standard when the expertise of the centers is considered.

Some patients with locally advanced tumors were primarily treated by surgery, whereas the current guidelines recommend neoadjuvant chemoradiotherapy.^9^ Even if based on European practices, this may lead to some criticism. However, the same observation has been published very recently based on a French nationwide population.^20^ This may be explained by the 11-year study period, with a high level of evidence for the benefit of neoadjuvant chemoradiotherapy only recently reported and implemented in clinical practice.^7^ This highlights how important it is for a specialized multidisciplinary team meeting in a center with high surgical volume to offer patients the optimal treatment plan at the time of diagnosis.

The risk of local nodal invasion increases with increasing depth of tumor invasion (T stage) because the lymphatic drainage of the esophagus is predominantly submucosal. Dubecz et al.^21^ showed in a large national series that the risk of nodal invasion in T1a esophageal cancer ranged from 6.4 to 9.5%, which increased to 19.6–22.9% for T1b tumors. We also showed that the incidence of node-positive disease in patients with clinical T2N0 disease receiving primary surgery was 50%.^22^ The results of this study also highlight the critical importance of accuracy in establishing advanced clinical T stage to identify patients at risk for pathologic nodal positive disease and a poor prognosis.

Although all the centers included in the French Eso-Gastric Tumors (FREGAT) group were experienced esophageal cancer centers, it must be acknowledged that 40% of the patients staged as clinical N0 went on to have pathologic N+ at surgery, highlighting the ongoing challenges with accuracy of clinical staging. Furthermore, an unexpected finding was the correlation of lower-third tumor location with pathologic nodal positivity, which may be secondary to the challenges of accurate performance and assessment of nodal spread using endoscopic ultrasound for distal esophageal tumors.

The association of poor tumor differentiation with pathologic nodal positivity also was demonstrated. However this raises the additional importance of good clinical staging endoscopy by experienced endoscopists with adequate biopsies to establish tumor differentiation accurately.

The results of this present study suggest that lymph nodes that are positive but remain undetected clinically (cN0pN+) have the same prognosis as those detected clinically before surgery (cN+pN+). This implies that lymph node size allowing radiologic detection is not a prognostic indicator influencing survival.

The current study clearly demonstrated the prognostic importance of nodal positivity, with a 5-year survival rate of approximately 20% for patients with pathologic nodal positivity who received surgery alone. Similar to previous publications, we demonstrated the lack of survival benefit seen with adjuvant chemotherapy for lymph node-positive patients.^23^ However, neoadjuvant therapy commonly results in downstaging of esophageal cancer and has been shown to improve survival in several randomized controlled trials.^4^^–^ 8 Nevertheless, the presence of positive lymph nodes after neoadjuvant therapy has been shown to confer a poor prognosis in terms of long-term survival.^24^,^25^ Thus, although the results of this study suggest that patients who have clinical N0 disease with risk factors for pathologic positivity should receive neoadjuvant therapy, it must be noted that a portion of these patients will fail to respond. Therefore, an important area of future research remains, namely, identification of responders to neoadjuvant therapy so a patient- and tumor-tailored approach to the management of esophageal cancer can be provided.

Interestingly, the cN+pN0 patients had an intermediate prognosis between that of the cN0pN0 and cN+pN+ patients. Because only patients treated with primary surgery were included in the study, the mechanism of this intermediate prognosis remains unclear, but may be a reflection of differences in pT stage or nodal clearance at surgery. This highlights the fact that even if the prognosis of esophageal cancer is primarily driven by pathologic lymph node invasion, clinical lymph node involvement also has a prognostic role to be taken into account when neoadjuvant treatment is considered, as well as clinical T stage classification.

## Conclusion

This large multicenter retrospective European study showed that cN0pN+ has a prognosis similar to that of cN+pN+ and substantially worse than that of cN0pN0. Patients with clinical N0 disease but risk factors for pathologic N+ disease, including advanced T stage, lower third tumor location, and poor tumor differentiation, may benefit from neoadjuvant therapy before surgery.

## Appendix 1: Adjusted Survival Analyses According to the Histologic Subtype and Clinical T Stage

### Squamous Cell Carcinoma

Subset analysis of the squamous cell carcinoma histologic subtype showed that cN0pN+ had a significantly reduced 5-year overall survival (hazard ratio [HR] 2.36; 95% confidence interval [CI] 1.82–3.07; *P* < 0.001) and event-free survival (HR 2.04; 95% CI 1.60–2.62; *P* < 0.001) compared with cN0pN0. However, cN0pN+ showed no significant difference in 5-year overall survival (HR 0.80; 95% CI 0.61–1.05; *P* = 0.108) or event-free survival (HR 0.85; 95% CI 0.64–1.11; *P* = 0.226) compared with cN+pN+.

### Adenocarcinoma

Similarly, subset analysis of the adenocarcinoma histologic subtype showed that cN0pN+ had a significantly reduced 5-year overall survival (HR 4.08; 95% CI 3.05–5.48; *P* < 0.001) and event-free survival (HR 4.10; 95% CI 3.08–5.45; *P* < 0.001) compared with cN0pN0. Again, cN0pN+ showed no significant difference in 5-year overall survival (HR 1.06; 95% CI 0.84–1.34; *P* = 0.608) or event-free survival (HR 0.91; 95% CI 0.73–1.14; *P* = 0.433) compared with cN+pN+.

### Clinical T1 and T2 Stages

For clinical T1 stage, cN0pN+ had a significantly reduced 5-year overall survival (HR 3.46; 95% CI 2.32–5.16; *P* < 0.001) and event-free survival (HR 3.22; 95% CI 2.19–4.74; *P* < 0.001) compared with cN0pN0. Compared with cN+pN+, cN0pN+ showed no significant difference in 5-year overall survival (HR 1.80; 95% CI 0.75–4.34; *P* = 0.191) or event-free survival (HR 1.87; 95% CI 0.78–4.50; *P* = 0.163).

Subset analysis of clinical T2 stage showed that cN0pN+ had a significantly reduced 5-year overall survival (HR 3.30; 95% CI 2.34–4.65; *P* < 0.001) and event-free survival (HR 3.08; 95% CI 2.22–4.27; *P* < 0.001) compared with cN0pN0. Compared with cN+pN+, cN0pN+ showed no significant difference in 5-year overall survival (HR 1.09; 95% CI 0.78–1.53; *P* = 0.606) or event-free survival (HR 1.01; 95% CI 0.73–1.40; *P* = 0.951).

### Clinical T3 and T4 Stage

Subset analysis of clinical T3 stage showed that cN0pN+ had a significantly reduced 5-year overall survival (HR 1.68; 95% CI 1.13–2.50; *P* = 0.011) and event-free survival (HR 1.66; 95% CI 1.13–2.44; *P* = 0.010) compared with cN0pN0. Compared with cN+pN+, cN0pN+ showed no significant difference in 5-year overall survival (HR 0.81; 95% CI 0.61–1.08; *P* = 0.147) or event-free survival (HR 0.81; 95% CI 0.62–1.07; *P* = 0.139).

For clinical T4 stage, cN0pN+ had a significantly reduced 5-year overall survival (HR 3.09; 95% CI 2.02–4.73; *P* < 0.001) and event-free survival (HR 2.50; 95% CI 1.68–3.71; *P* < 0.001) compared with cN0pN0. Compared with cN+pN+, cN0pN+ showed no significant difference in 5-year overall survival (HR 1.01; 95% CI 0.69–1.48; *P* = 0.953) or event-free survival (HR 0.78; 95% CI 0.54–1.13; *P* = 0.189).

## Appendix 2: Collaborators List

Department of Digestive Surgery Bordeaux, France: Guillaume Luc, MD. Department of Thoracic Surgery Bordeaux, France: Magalie Cabau, MD; Jacques Jougon MD, PhD. Department of Digestive Surgery, Brest, France: Bogdan Badic, MD; Patrick Lozach, MD, PhD. Department of Digestive Surgery, Brussels ULB Erasme Bordet University, Brussels, Belgium: Issam El Nakadi, MD, PhD; Serge Cappeliez, MD, PhD. Department of Digestive Surgery, Caen, France: Gil Lebreton, MD; Jean Lubrano, MD, PhD; Arnaud Alves, MD, PhD. Department of Digestive Surgery, Clermont-Ferrand, France: Renaud Flamein, MD; Denis Pezet, MD, PhD. Department of Digestive Surgery, Louis Mourier University Hospital, Paris, France: Federica Pipitone, MD; Bogdan Stan Iuga, MD; Nicolas Contival, MD; Eric Pappalardo, MD. Department of Digestive Surgery, Lausanne University Hospital, Lausanne, Switzerland: Styliani Mantziari, MD; Nicolas Demartines, MD, PhD. Department of Digestive Surgery, Lille, France: Flora Hec, MD; Marguerite Vanderbeken, MD; Sébastien Degisors, MD; Hélène Marin, MD. Department of Digestive Surgery, Limoges, France: Fabien Fredon, MD; Alain Gainant, MD; Muriel Mathonnet, MD, PhD. Department of Digestive Surgery, Croix Rousse University Hospital, Lyon, France: Jean-Marc Bigourdan, MD; Salim Mezoughi, MD; Jean-Yves Mabrut, MD, PhD; Christian Ducerf, MD. Department of Digestive Surgery, Edouard Herriot University Hospital, Lyon, France: Oussama Baraket, MD; Gilles Poncet, MD. Department of Digestive Surgery, Lyon Sud University Hospital, Lyon, France: Delphine Vaudoyer, MD; Peggy Jourdan Enfer, MD; Laurent Villeneuve, MD; Olivier Glehen, MD, PhD. Department of Digestive Surgery, Montpellier, France: Thibault Coste, MD; Jean Michel Fabre, MD, PhD. Department of Digestive Surgery, Institut de Cancérologie de Lorraine, Nancy, France: Frédéric Marchal, MD. Department of Digestive Surgery, Nancy, France: Romain Frisoni, MD; Ahmet Ayav, MD, PhD; Laurent Brunaud, MD, PhD; Laurent Bresler, MD, PhD. Department of Thoracic Surgery, Nice, France: Charlotte Cohen, MD; Olivier Aze, MD; Nicolas Venissac, MD; Daniel Pop, MD; Jérôme Mouroux, MD. Department of Digestive Surgery, Nîmes, France: Ion Donici, MD; Michel Prudhomme, MD, PhD. Department of Digestive Surgery, Pitié Salpétrière University Hospital, Paris, France: Emanuele Felli, MD; Stéphanie Lisunfui, MD; Marie Seman, MD; Gaelle Godiris Petit, MD; Mehdi Karoui, MD, PhD; Christophe Tresallet, MD, PhD; Fabrice Ménégaux, MD, PhD. Department of Digestive Surgery, Lariboisière University Hospital, Paris, France: Brice Malgras, MD; Denis Lantuas, MD; Karine Pautrat, MD; Marc Pocard, MD, PhD.
